# Diabetes: Recent Advances and Future Perspectives

**DOI:** 10.3390/biomedicines12122875

**Published:** 2024-12-18

**Authors:** Miodrag Janić, Andrej Janež, Mohamed El-Tanani, Viviana Maggio, Manfredi Rizzo

**Affiliations:** 1 Department of Endocrinology, Diabetes and Metabolic Diseases, University Medical Centre Ljubljana, 1000 Ljubljana, Slovenia; andrej.janez@kclj.si; 2 Faculty of Medicine, University of Ljubljana, 1000 Ljubljana, Slovenia; 3 School of Medicine, PROMISE Department of Health Promotion Sciences Maternal and Infantile Care, Internal Medicine and Medicinal Specialties, University of Palermo, 90133 Palermo, Italy; viviana.maggio01@unipa.it (V.M.);; 4 College of Pharmacy, Ras Al Khaimah Medical and Health Sciences University, Ras Al Khaimah P.O. Box 11127, United Arab Emirates; eltanani@rakmhsu.ac.ae

Diabetes is a chronic metabolic disorder distinguished by persistent hyperglycemia. It constitutes an important and growing global health concern and is among the most prevalent metabolic disorders. As shown in the 2021 IDF Diabetes Atlas by the International Diabetes Federation [1], the estimated worldwide prevalence was approximately 537 million individuals, representing 10.5% of the adult population. Projections suggest that by 2045, this number will increase to 783 million. In particular, three-quarters of adults diagnosed with diabetes reside in low- to middle-income countries. Furthermore, diabetes was responsible for 6.7 million deaths in 2021. Type 2 diabetes is identified as the most common form of diabetes, comprising 90% of cases worldwide [1]. Among people with type 2 diabetes, approximately 32–35% develop cardiovascular disease and approximately one-third of all cases were due to atherosclerotic cardiovascular disease, primarily coronary artery and cerebrovascular diseases [2,3,4].

Management strategies for people with diabetes have been improved, leading to a reduction in overall mortality by approximately a third in the past two decades. However, this decrease in mortality is also observed in individuals without diabetes, which maintains the disparity in mortality rates between those with and without the disease. Additionally, the predominant causes of mortality have changed, with cancer replacing cardiovascular-related causes [5]. However, an earlier diagnosis of type 2 diabetes is associated with an increased number of years of life lost and a higher risk of mortality due to complications related to diabetes [6]. Diabetes continues to be the leading cause of blindness and kidney failure that warrants dialysis worldwide and is also a significant contributor to non-traumatic amputations [7]. Taking into account these concerning data, ongoing efforts are being made to improve the management strategies for people with diabetes.

Although the treatment of type 1 diabetes requires compulsory insulin therapy, the management of type 2 diabetes has transitioned from a glucose-centric approach to a more heterogeneous multifactorial strategy, the efficacy of which was already demonstrated in the STENO-2 trial, even in the absence of beneficial cardiovascular antihyperglycemic therapy. Today, in the context of type 2 diabetes, a person-centered approach based on pillars is used to simultaneously address diabetes-related complications and comorbidities. These pillars are based on nonpharmacological interventions, such as dietary modifications and physical activity, but also physical behaviors at 24 h (involving sitting/breaking up prolonged sitting, stepping, sleep, sweating, and strengthening). The treatment of cardiovascular risk factors, including smoking cessation, control of arterial hypertension, and lipid management, is equally important. In the context of pharmacological therapy, the first pillar includes atherosclerotic cardiovascular disease or indicators of high risk in individuals, heart failure, and chronic kidney disease. These conditions are known to perpetuate each other, requiring an assertive approach to their management using medications with documented cardiovascular and renal benefits. The preferred pharmacological agents are sodium-glucose cotransporter-2 (SGLT-2) inhibitors and glucagon-like peptide-1 (GLP-1) receptor agonists, which should be used regardless of glycemic control and regardless of previous use of metformin [8,9]. The second pillar is related to glycemic control, where, together with the first pillar, priority is given to potent and efficacious antihyperglycemic drugs, with an emphasis on their safety [10]. The third and final pillar underscores the importance of achieving and maintaining weight management, prioritizing the most effective and potent therapies for weight reduction [11].

Glycemic control is incorporated as a supplementary aspect of this pillar-based approach, indicating that the incorporation of effective and safe therapies should be undertaken as the disease advances and demands emerge, while consistently ensuring adherence to the pillar-based objectives. These initiatives culminate in a reduction in complications and, most importantly, an improvement in quality of life [12]. It can be noted that, even before the recommendations of diabetologists, cardiologists advocated for the early use of SGLT-2 inhibitors and GLP-1 receptor agonists in drug-naïve individuals with type 2 diabetes who have established atherosclerotic cardiovascular disease or are at high/very high cardiovascular risk (characterized by target organ damage or multiple risk factors) [13].

The rationale for this approach is based on the large amount of data collected since 2015, following the initial publication of the cardiovascular superiority of empagliflozin in people with type 2 diabetes [14]. Furthermore, the accumulation of data persistently supports and reinforces the imperative to pursue this therapeutic strategy with greater rigor [15]. Thus, it is concerning that even in high-income countries, the people who would most benefit from this treatment are not properly administered [16]. The data continue to accumulate. Most recently, the SOUL study was reported, which demonstrated that oral semaglutide achieved a 14% reduction in major adverse cardiovascular events (MACEs) compared to placebo in a trial involving 9650 individuals with type 2 diabetes and established cardiovascular disease and/or chronic kidney disease, and 49% of the trial participants had used SGLT-2 inhibitors at some point during the trial [11]. This development aligns with the continuum demonstrated by GLP-1 receptor agonists, whose anti-atherosclerosis mechanism of action is highly anticipated through the outcomes of the Semaglutide Anti-Atherosclerotic Mechanisms of Action Study (SAMAS) [17].

Regarding SGLT-2 inhibitors, their beneficial cardiovascular protective effects continue to be validated. A recent study involving 36,670 eligible initiators of empagliflozin and 20,606 eligible initiators of dapagliflozin with type 2 diabetes demonstrated that the absolute risk of MACE at 6 years was equivalent between both groups of users. These findings were consistent between individuals with established atherosclerotic cardiovascular disease and those without; furthermore, no differences were observed between individuals with or without heart failure [18]. The findings suggest a class effect, thus necessitating intervention and the appropriate prescription of these medications, particularly in accordance with harmonized consensuses and guidelines, which are converging in recommendations on a global scale.

The pillar-based approach can be enhanced by the addition of an innovative therapeutic agent, the non-steroidal mineralocorticoid receptor antagonist finerenone, which is designed for individuals with type 2 diabetes who exhibit at least a moderately increased level of albuminuria. Integration of an SGLT-2 inhibitor and GLP-1 receptor agonist with finerenone has shown a 35% reduction in the risk of MACE compared to conventional treatment modalities. Over a three-year period of therapy, this corresponds to an absolute risk reduction of 4.4% and a number needed to treat of 23. This can be further interpreted as an extension of 3.2 years of event-free survival for MACE, 3.2 years of event-free survival with respect to hospitalization for heart failure, 5.5 years of delay in the progression of chronic kidney disease, 2.2 years of event-free survival of cardiovascular mortality, and 2.4 years of event-free survival of all-cause mortality [19].

However, there is a significant unmet need among individuals with type 1 diabetes, who continue to receive limited benefit from these categories of pharmacotherapy. Predominant safety concerns, especially the risk of diabetic ketoacidosis, have cast considerable doubt on its application in this demographic. However, it is important to recognize that these individuals are likely to derive advantages from such treatments. In recent years, a noticeable trend of increased off-label prescriptions has been observed, to mitigate cardiovascular and renal risks [20]. Numerous analyses and meta-analyses have demonstrated the advantageous effects of these treatments in this population of patients. A recent report indicated a substantial reduction in albuminuria, with an average difference of 23% observed in people with type 1 diabetes who received SGLT-2 inhibitors, regardless of the type or dose administered [21].

In addition to the cardiometabolic and renal implications of contemporary antidiabetic treatments, the importance of direct glucose regulation should not be overlooked. It is well established that the early and intensive management of hyperglycemia is associated with a reduced risk of microvascular and potentially macrovascular complications in people with diabetes. Consequently, from the time of diagnosis, a targeted focus should be on implementing early, rigorous, and precise glucose control measures. This effect, called the legacy effect or metabolic memory on macrovascular complications, has been validated by a recent Italian registry trial in which 251,339 people, newly diagnosed without cardiovascular disease and with type 2 diabetes, were stratified based on average glycated hemoglobin levels (HbA1c) during the initial 12, 24, and 36 months after diagnosis. Subsequently, the trial tracked the incidence of cardiovascular disease in the following years. The HbA1c values for each of the three stratification periods were classified as <5.7%, 5.7–6.4%, 6.5–7.0%, 7.1–8.0%, and >8.0%. This study assessed the associations between glycemic control and the onset of cardiovascular disease. A mean HbA1c of <5.7% was used as a reference standard. All groups that exceeded this threshold were found to have an increased risk of cardiovascular disease. Specifically, 1-year exposure after diagnosis to HbA1c levels >5.7% resulted in an increase in cardiovascular risk of 24%, 42%, 49%, and 56%, corresponding to the HbA1c categories [22]. The same researchers indicate that the early administration of SGLT-2 inhibitors in the first 2 years after diagnosis mitigates this association with increased risk, supporting the rationale for the prompt utilization of these medications after the diagnosis of type 2 diabetes [23].

These findings contrast with the prevalent belief that glycemic control is not particularly relevant for the prevention of cardiovascular disease in type 2 diabetes. Currently, numerous glucose-lowering medications do not induce hypoglycemia, leading to a reevaluation of appropriate glucose targets in many patients. This study demonstrated that, within routine care, the early attainment and maintenance of HbA1c targets within the normal range (HbA1c < 5.7%) are feasible and advantageous for the prevention of cardiovascular and microvascular disease [22]. Therefore, it is essential to re-evaluate our current perspectives and strive for normoglycemia at the beginning of a diagnosis of diabetes. A sub-analysis of the SURPASS 1–4 trials, which evaluated 3229 people with type 2 diabetes, revealed that after 40 weeks of treatment with the first glucose-dependent insulinotropic peptide/GLP-1 (GIP/GLP-1) dual co-agonist, tirzepatide, 44% of the participants achieved HbA1c levels of 5.7–6.5%, while 37% reached HbA1c levels below 5.7%. Normoglycemia was achieved along with the highest mean weight loss of 14.1% at week 40, and a mean reduction in HbA1c of 2.96%. Factors that predispose people to achieve normoglycemia included younger age, a shorter duration of diabetes, lower initial levels of HbA1c, and prior use of metformin alone. Furthermore, the reduction in cardiometabolic risk factors was significant, with decreases in both systolic and diastolic blood pressure, in addition to triglyceride levels. Gastrointestinal side-effects were reported as expected, but among individuals achieving normoglycemia, 98.5% did not experience level 2 or 3 hypoglycemia [24,25]. The aforementioned evidence further supports the need to address the hesitancy and reluctance toward the aggressive intensification of antihyperglycemic therapy in routine clinical practice. Effective interventions, such as potent medications with beneficial outcomes, must be implemented from the beginning of treatment.

Tirzepatide, despite the lack of conclusive evidence from the cardiovascular outcome trial, SURPASS-CVOT, has demonstrated significant beneficial effects on mortality, adverse cardiovascular events, and renal outcomes in individuals with type 2 diabetes, as indicated by a recent analysis by Chuang et al. In this retrospective study, 14,834 people with type 2 diabetes started treatment with tirzepatide, while 125,474 people started treatment with GLP-1 receptor agonists. Following a median follow-up period of 10.5 months, tirzepatide was associated with a 42% relative reduction in all-cause mortality, a 20% relative reduction in MACE, a 48% relative reduction in renal events, and a 46% relative reduction in major adverse renal events. Treatment with tirzepatide also resulted in a greater reduction in HbA1c and body weight compared to GLP-1 receptor agonists [26].

The encouraging prospects for the management of diabetes are not merely attributed to the substantial evidence on the beneficial effects of antihyperglycemic medications, although it is very clear that the early and proper use of these agents is imperative in reducing complications and improving quality of life. There is also an active development pipeline of novel treatments that target additional pathways involved in the pathogenesis of diabetes. The advent of once-weekly insulin administration is imminent and is expected to transform the domain of diabetes care. Insulin icodec has been approved for use within the European Union and phase 3 clinical trials with efsitora alpha are currently in progress. These new forms of insulin, known for their efficacy and safety, are expected to address the therapeutic inertia often associated with insulin initiation and improve the adherence to treatment protocols among patients. Furthermore, we are witnessing an era in which the onset of clinically apparent type 1 diabetes could be postponed with the use of teplizumab. Personalized medicine, particularly in the context of diabetes, is gaining traction, as it allows the development of customized treatment plans based on individual genetic profiles and clinical characteristics. Artificial intelligence tools will help by predictive modeling to assess disease progression and risk. In addition, artificial intelligence has penetrated the field of diabetes more extensively than anticipated, through technological advancements such as closed-loop insulin delivery systems, which increasingly emulate a potential artificial pancreas. Continuous glucose monitoring systems are also becoming accessible to a wider range of people with various types of diabetes, creating opportunities for innovative approaches to diabetes management. The potential contributions of stem cell therapy to diabetes, particularly in the realm of regenerative medicine for beta cell replacement, remain to be fully elucidated.

Lastly, but of significant importance, global disparities persist in diabetes care and access to treatment. Addressing these disparities and improving awareness and education about diabetes in low- and middle-income countries will remain critical challenges in the effort to combat this debilitating disease.
